# Chaperone-Mediated Autophagy and Mitochondrial Homeostasis in Parkinson's Disease

**DOI:** 10.1155/2016/2613401

**Published:** 2016-06-16

**Authors:** Ruixin Yang, Guodong Gao, Zixu Mao, Qian Yang

**Affiliations:** ^1^Department of Neurosurgery, Tangdu Hospital, The Fourth Military Medical University, 569 Xinsi Road, Xi'an, Shaanxi 710038, China; ^2^Department of Pharmacology and Neurology, School of Medicine, Emory University, Atlanta, GA 30322, USA

## Abstract

Parkinson's disease (PD), a complex neurodegenerative disorder, is pathologically characterized by the formation of Lewy bodies and loss of dopaminergic neurons in the substantia nigra pars compacta (SNc). Mitochondrial dysfunction is considered to be one of the most important causative mechanisms. In addition, dysfunction of chaperone-mediated autophagy (CMA), one of the lysosomal proteolytic pathways, has been shown to play an important role in the pathogenesis of PD. An exciting and important development is recent finding that CMA and mitochondrial quality control may be linked. This review summarizes the studies revealing the link between autophagy and mitochondrial function. Discussions are focused on the connections between CMA and mitochondrial failure and on the role of MEF2D, a neuronal survival factor, in mediating the regulation of mitochondria in the context of CMA. These new findings highlight the need to further explore the possibility of targeting the MEF2D-mitochondria-CMA network in both understanding the PD pathogenesis and developing novel therapeutic strategies.

## 1. Introduction

Parkinson's disease (PD) is the second most common neurodegenerative disorder after Alzheimer's disease. It affects about 1% of people above 60 years of age [1]. Rigidity, bradykinesia, postural instability, and tremors are the four characteristic clinic features of PD. Parkinson's disease is caused by the loss of dopaminergic neurons in the substantia nigra pars compacta (SNc), which leads to decreased dopamine (DA) levels in the dorsal striatum [2, 3]. Pathologically, the cytoplasm of SNc dopaminergic neurons in the brain of PD patients is characterized with the presence of Lewy bodies, which represents aggregation of proteins including *α*-synuclein [4]. Current treatment available for PD offers only symptomatic relief but cannot modify the disease progression. Nor can it slow down the progress of the disease. Although over 90% of PD cases are sporadic, the remaining 10% have a family inheritance [5]. Mutations in PARK 1 (SNAC) and PARK 8 (LRRK2) cause autosomal-dominant PD, while mutations in PARK2 (PARKIN), PARK6 (PINK1), and PARK7 (DJ-1) are responsible for autosomal recessive PD [5]. Neurons in both familial and sporadic PD cases display the same key pathophysiological features. Extensive research on PD pathogenesis has firmly established mitochondrial dysfunction, oxidative stress, and impaired protein clearance as the key cellular processes altered in both familial and sporadic PD [6–8]. Interestingly, mutations in PARKIN, PINK1, and DJ-1 all lead to mitochondrial dysfunction [9, 10].

Growing evidence shows recently that autophagy, a protein clearance pathway, is critical for maintaining mitochondrial homeostasis and is impaired in neurodegenerative disorders such as PD [11, 12]. Autophagy is a cellular self-eating process in which lysosomes degrade intracellular components including proteins and other organelles. Autophagy is active under the normal basal metabolic condition as well as activated upon stress such as starvation, both of which are important to maintain cellular homeostasis [13]. There are three types of autophagy, microautophagy, macroautophagy, and chaperone-mediated autophagy (CMA). These processes involve different mechanisms and may serve different cellular functions [14, 15]. Both macroautophagy, which is termed mitophagy when targeting mitochondria, and CMA are associated with mitochondrial function [16–20]. Many studies indicate that autophagy level is often downregulated in various neurodegenerative diseases, such as Alzheimer's disease (AD), Huntington's disease (HD), and amyotrophic lateral sclerosis (ALS) [21–23], and impaired autophagic function may in varying degree contribute to the neurodegenerative process. For PD, increasing evidence shows that dysfunction of mitophagy and CMA appears to be particularly prominent and a key pathogenic theme common to multiple genetic risk factors associated with the disease [9, 17, 24, 25].

## 2. Mitophagy and Parkinson's Disease

Mitophagy selectively removes damaged mitochondria. The process of mitophagy needs two key proteins, PINK1 and Parkin. The genes encoding these two proteins are mutated in autosomal recessive parkinsonism [9], supporting the idea that dysfunction of mitophagy caused by PINK1 and PARKIN mutation may be an important mechanism in PD pathogenesis.

PINK1 is a key protein in the pathway of mitophagy [9]. Structurally, the C terminus of PINK1 is predicted to be a kinase domain while its N terminus contains a mitochondrial targeting sequence, which helps it locate onto mitochondrial outer membrane and transfers it into mitochondria [26]. PINK1 accumulates specifically on dysfunctional mitochondria and recruits Parkin from the cytosol [27, 28]. Parkin is an E3 ubiquitin ligase with an N-terminal ubiquitin-like domain and a C-terminal ubiquitin ligase domain [29, 30]. After being recruited to the damaged mitochondria, Parkin ubiquitinates substrates on the damaged mitochondria to instigate their elimination by autophagy [31, 32]. Mutations in both PINK1 and Parkin reduce their ability to eliminate dysfunctional mitochondria, indicating that PINK1 and Parkin are essential for mitochondrial quality control [33–35]. Accumulation of dysfunctional mitochondria causes stress to the SNc DA neurons. A reduction in mitophagic activity following a loss of either PINK1 or Parkin function may trigger or exacerbate the loss of homeostasis and viability of these neurons, contributing to PD pathogenesis [36–39].

Although mitophagy plays a crucial role in mitochondria quality control, it can only recognize mitochondria that have already been damaged and remove them. PINK1 and Parkin also regulate mitochondria via nonmitophagy processes. Mitochondria are cellular respiratory factory and have a high oxidative level. This oxidative environment can often damage individual mitochondrial proteins without causing irreparable mitochondrial damage. In such cases, it is best for cells to dispose the damaged individual proteins via proteolytic pathways to maintain mitochondrial homeostasis without the need for mitochondria removal and biogenesis. PINK1 and Parkin appear to also participate in these pathways. For example, an interesting in vivo study showed that the turnover of many mitochondrial respiratory chain subunits is impaired when Parkin and PINK1 are mutated [40]. Furthermore, this impairment caused by Parkin mutation seems to be greater than it is in Atg7 mutant. This finding cannot be simply explained by the model that Parkin acts upstream of Atg7 to promote mitophagy. Instead, it is more consistent with the notion that in addition to mitophagy PINK1-Parkin pathway may promote the selective turnover of mitochondrial proteins such as the respiratory complex subunits [40]. PINK1 and Parkin are also related to the biogenic pathway of mitochondria derived vesicles (MDVs), in which vesicles of budding off mitochondria with a specific repertoire of cargo proteins are ultimately targeted to lysosomes for degradation [41]. In this study, Parkin colocalizes with MDVs in a PINK1-dependent manner and stimulates their formation in response to antimycin A, a potent generator of reactive oxygen species (ROS). And once formed, the MDVs target to lysosomes for degradation in a manner independent of canonical mitophagy. These findings implicate that PINK1 and Parkin participate in a mitochondrial quality control pathway besides mitophagy. Moreover, Parkin mediates the ubiquitination of some outer mitochondrial membrane (OMM) proteins such as Mitofusins (Mfns) and Miro1 to regulate their function or degradation by proteasome [9, 42]. PINK1 may also be involved in this process by acting as ubiquitin kinase. Through Mfns, PINK1 and Parkin can potentially regulate mitochondrial fission and fusion.

## 3. CMA and Cellular Homeostasis in Parkinson's Disease

Chaperone-mediated autophagy, one of the lysosomal proteolysis pathways, is characterized by its specificity in selective degradation of substrate proteins. This process can be divided into four steps: (1) recognizing substrate proteins and targeting them to the lysosomes; (2) binding to lysosomal receptor and unfolding of substrate proteins; (3) translocating substrates into lysosomes; and (4) degrading substrates in the lysosomal lumen [25]. The substrate proteins of CMA are recognized in the cytosol by the chaperone protein heat shock-cognate protein of 70 KDa (Hsc70) via a pentapeptide motif similar to KFERQ in sequence [43, 44]. This motif is not in strict conformance with a specific amino acid residue sequence but is like a pattern recognition motif related to the charge and hydrophobicity of amino acid residues [45, 46]. Posttranslational modifications such as phosphorylation and acetylation can facilitate an imperfect motif acquiring more effective recognition [47–49]. It is predicted that almost 30% of cytosolic proteins have a KFERQ-like motif, but only a few of them have been experimentally confirmed as CMA substrates [50].

Once recognized by Hsc70, the substrate is targeted to the surface of lysosomal membrane and bound to the cytosolic tail of the lysosome-associated membrane protein type 2A (LAMP2A) [51]. LAMP2A exists as monomer at the lysosome membrane. During CMA, it oligomerizes to form a multiprotein complex to facilitate the translocation of substrates into the lysosomal lumen [52]. Before substrate protein translocation, it needs to be unfolded. This is mediated by Hsc70 and its co-chaperones [53]. Translocation of the substrate proteins into the lysosomal lumen requires a lysosomal resident form of Hsc70 (lys-Hsc70) [54]. The mechanism by which lys-Hsc70 facilitates substrate protein translocation remains unknown. After substrate translocation, LAMP2A rapidly disassembles from the translocational multimer-complex into monomers to which substrates can bind again [52]. Thus, the rate of CMA is modulated by the level of LAMP2A and the rate of assembly/disassembly of the translocation complex [25].

With its highly selective mechanism, CMA is especially suited for removing misfolded, oxidized, or damaged cytosolic proteins under both physiological and pathological conditions [50, 55]. This removal is not only an amino acid recycling pathway, but also a mechanism of balancing cellular homeostasis. Under moderate oxidative stress, which is related to and can cause mitochondrial dysfunction, CMA is often activated to accelerate the elimination of proteins damaged by oxidative stress [56]. Consequently, blocking the upregulation of CMA under those conditions leads to the accumulation of proteins damaged by oxidative stress and impairs mitochondrial function and cellular viability [57]. CMA is also activated under other stress conditions such as exposure to prolonged starvation [58] and hypoxia [59]. It appears that CMA is an essential stress response mechanism that is required to maintain cellular homeostasis through the removal of damaged proteins under various conditions.

There are multiple lines of evidence for the impairment of CMA activity in both familial and sporadic PD [60, 61]. Two critical proteins mutated in familial PD, *α*-synuclein and leucine-rich repeat kinase 2 (LRRK2), are both degraded by lysosomes via CMA [62–64]. *α*-synuclein is a key factor in PD pathogenesis. The accumulation of both wild-type and mutant *α*-synuclein, which is caused by the dysfunction of autophagy-lysosome pathways including macroautophagy and CMA, causes SNc neuron loss [64–66]. One proposed pathogenic mechanism by which mutant *α*-synuclein may exert stress is interference of cellular protein homeostasis through the blockade of CMA process [62]. Mutation in LRRK2 is the most common cause of familial PD. Although wild-type LRRK2 itself is degraded by CMA, the most common pathogenic mutant form of LRRK2, G2019S, is poorly degraded by this pathway. Moreover, LRRK2 mutants or high levels of WT LRRK2 bind to the lysosomal membrane and inhibit the assembly of the CMA translocation complex [63]. Furthermore, mutation of UCHL1, which is also associated with familial PD, has also been shown to inhibit CMA process [67, 68]. Thus, CMA controls the turnover of several proteins whose mutation is linked to familial PD. Inhibition of CMA appears to be a key common mechanism through which multiple proteins associated with familial PD exert their toxic effect.

## 4. MEF2D, CMA, and Oxidative Stress in Parkinson's Disease

Myocyte enhancer factor 2 (MEF2) is initially identified as a transcription factor vital for muscle cell differentiation [69]. There are four isoforms of MEF2, MEF2A-MEF2D. MEF2s share a highly homologous sequence of the first 86 amino acids at the N-terminus, which participates in MEF2s hetero- or homodimerization and their binding to an A/T rich cis-acting DNA element. The C-terminal sequence of MEF2s is diverse and responsible for MEF2-mediated transcription activation. MEF2s can be modulated by posttranslational modifications such as phosphorylation at the C-terminus and interaction with other cofactors [70].

Although MEF2s are first identified in muscle cells, recent studies have revealed that MEF2s play an important role in several cellular pathways in neurons including neuronal survival [70–72]. It has been shown that neuronal activity activates MEF2 by p38-mediated phosphorylation. Inhibition of MEF2s blocks the neuronal activity-induced survival of cerebellar granule neurons, leading to their apoptosis.

The connection between MEF2D and Parkinson's disease started with the finding that cyclin dependent kinase 5 (Cdk5) directly phosphorylates MEF2D at Ser444 under stress conditions [73], which leads to an impairment of MEF2D transcriptional function. It was shown subsequently that phosphorylation of MEF2D at Ser444 promotes its degradation by caspases, leading to a sharp reduction of MEF2D level and neuronal death [74]. It was late demonstrated that this Cdk5-mediated inhibition of MEF2D is involved in 1-methyl-4-phenyl-1,2,3,6-tetrahydropyridine- (MPTP-) induced loss of DA neurons in a mouse model of PD [75, 76]. Therefore, MPTP-induced loss of SNc DA neurons in vivo may be in part due to Cdk5-mediated modulation of MEF2D [77].

In addition to being modulated by Cdk5, MEF2D is also regulated by CMA [78]. MEF2D has several imperfect KFERQ-like motifs at the N-terminus, mediating its interaction with Hsc70 and degradation by CMA. The blockage of CMA leads to an increase of cellular MEF2D level, and the accumulated MEF2D shows a decrease in DNA binding ability. Thus, the inhibition of CMA significantly impairs MEF2D function. Furthermore, degradation of MEF2D by CMA can be blocked by both wild-type and mutant *α*-synuclein. Though efforts were made to understand how the increase in the level of wild-type *α*-synuclein causes PD, the pathogenic mechanisms underlying it toxicity remain unclear. The finding that increased level of wild-type *α*-synuclein interferes CMA-mediated degradation and homeostasis of MEF2D provides a mechanism by which aberrant increase in wild-type *α*-synuclein induces neuronal death.

SNc DA neurons show increased oxidative stress in PD. Oxidative stress is considered to be a key mechanism that either triggers or exacerbates the pathological process in PD. How oxidative stress modulates neuronal activity is an intense and important area of investigation. Recent studies show that a short-term exposure to 6-hydroxydopamine (6-OHDA), a neurotoxin used to model PD in rodents, leads to oxidization of MEF2D [79]. MEF2D can be oxidized at cysteine residues and carbonylated. Oxidized MEF2D loses its DNA binding ability as well as gene transcription control and is preferentially removed by CMA, which is activated by moderate oxidative stress as a protective response [79]. High levels of *α*-synuclein, neurotoxins, and excitotoxicity are known to cause excessive oxidative stress [80–82], which not only oxidizes MEF2D but also inhibits CMA. Therefore, the combination of losing both survival factor such as MEF2D and CMA protection may underlie, at least in part, the toxic effects of those diverse stress conditions. Oxidative stress is widely considered to be one of the key mechanisms that trigger or exacerbate the pathological process in PD [83–85]. Given the vital role of MEF2D in the survival of SNc DA neurons [78] and CMA-mediated protection, enhancing MEF2D or CMA should be explored further as a therapeutic strategy for PD.

## 5. MEF2D and Mitochondrial Function in Parkinson's Disease

MEF2s have been studied as a nuclear factor for its dynamic roles in many cellular processes. For example, previous research of MEF2A in muscle indicates that MEF2A regulates mitochondrial function through controlling nuclear gene expression [86]. However, this nuclear centric role of MEF2s has been expanded with the unexpected finding of MEF2 function outside the nucleus in mitochondria [87].

In an elegant study [87], researchers showed that a portion of MEF2D is localized in mitochondria in neuronal cells. This localization is mediated by the N-terminal 33 amino acid residues of MEF2D and requires mitochondria heat shock protein 70 (mtHsp70). Functionally, MEF2D regulates mitochondrial DNA (mtDNA) expression. mtDNA is a circular DNA, containing 16,569-base-pair-length genome that encodes 13 genes for subunit components of oxidative phosphorylation and its own tRNAs and rRNAs [88]. It has a heavy (H) strand and a light (L) strand determined by buoyant densities. The L strand encodes a single polypeptide, NADH dehydrogenase 6 (ND6), an essential component of complex I [89, 90]. Mutations in the* ND6* gene or changes of its transcriptional level are associated with PD [91, 92]. Mitochondrial MEF2D binds to mtDNA in the coding region of the* ND6* gene through a MEF2 site (5′-C^73^CTATTTATG^82^-3′) to directly control the transcription of* ND6* gene [87]. Inhibition of mitochondrial MEF2D activity reduces the levels of ND6 mRNA and protein. Reduction of ND6 level, triggered by the loss of mitochondrial MEF2D function, decreases complex I level and activity, reduces the level of ATP, and increases the level of H_2_O_2_. These findings demonstrate that mitochondrial MEF2D directly regulates ND6 and affects mitochondrial function. Increasing mitochondrial MEF2D level promotes the survival of SNc DA neurons under MPTP-induced toxicity. In both postmortem PD patient brains and MPTP model of PD, MEF2D colocalization with mitochondria is reduced, which correlates with and accounts for the reduced transcription of* ND6* gene in these specimens.

Complex I deficiency has been shown to occur in the mitochondria in PD [93, 94]. The discovery of MEF2D-ND6 axis demonstrates clearly that loss of mitochondrial MEF2D contributes to the mitochondrial dysfunction and may underlie part of the pathogenic process in PD. Since the level and activity of mitochondrial MEF2D are also regulated by oxidative stress and CMA [79], together, these findings provide another pathway by which CMA may closely modulate mitochondrial activity via regulation of MEF2D.

## 6. CMA and New Targets in Mitochondrial Homeostasis

As mentioned previously, CMA maintains cellular homeostasis in basal conditions through a highly selective protein degradation mechanism. It is predicted that almost 30% of cytosolic proteins have a KFERQ-like motif. Although only a few of them have been experimentally confirmed as CMA substrates, it highlights the huge potential for CMA being involved in regulating many important cellular processes. We investigated our previous unpublished data showing many possible CMA substrates are correlative with mitochondria function. By comparing the levels of proteins by mass spectrum analysis following up- or downregulation of LAMP2A, we identified the proteins whose levels changed sharply in a LAMP2A sensitive manner following activation of CMA. Among these proteins, a majority of them are involved in mitochondrial function (unpublished data). Furthermore, our analysis revealed that a decrease in CMA activity is correlated closely with a significant increase in the level of ROS and decrease in mitochondrial membrane potential. Identifying and validating the individual protein substrates of CMA that are directly involved in mediating these mitochondrial changes should provide us with mechanistic insights into how CMA specifically regulates mitochondrial homeostasis under both physiological and pathological conditions.

To this end, our recent work on regulation of DJ-1 by CMA provides such an example. DJ-1, also known as PARK7, is a mitochondria related protein that regulates the organellar function and morphology and antioxidative response [95]. Mutation of* DJ-1* gene leads to mitochondrial defects and is associated with autosomal recessive familial PD [96]. Our study showed that DJ-1 is a direct CMA substrate [97]. Moreover, CMA preferentially degrades the nonfunctional and oxidatively damaged DJ-1 and protects cells against neurotoxin-induced mitochondrial damage and stress. Thus, our identification of CMA in maintaining mitochondrial homeostasis via regulation of DJ-1 serves to highlight and strengthen the notion that there is a strong and critical link between CMA and mitochondria.

## 7. Conclusion

Mitochondrial dysfunction is an important cellular feature in PD pathogenesis. In addition to the evidence of mitophagy failure as a cause for mitochondrial damage, recent studies support a strong link between CMA and mitochondria and a role for loss of CMA activity in mitochondrial dysfunction.

CMA has the potential to regulate many cellular pathways and maintains cellular homeostasis. Furthermore, it protects cells from diverse stress conditions and promotes cellular viability, especially neurons. Although CMA has been shown to participate in some cellular processes, few studies have investigated whether and how CMA directly regulates mitochondrial function. Since CMA plays a vital role in protecting neurons from stress and SNc DA neurons are especially sensitive to mitochondrial dysfunction, it is essential for us to clarify the role of CMA in mitochondrial dysfunction in PD pathogenesis.
